# Effects of the Na^+^/H^+^ Ion Exchanger on Susceptibility to COVID-19 and the Course of the Disease

**DOI:** 10.1155/2021/4754440

**Published:** 2021-06-15

**Authors:** Medine Cumhur Cure, Erkan Cure

**Affiliations:** ^1^Department of Biochemistry, Private Kucukcekmece Hospital, Istanbul, Turkey; ^2^Department of Internal Medicine, Ota&Jinemed Hospital, Istanbul, Turkey

## Abstract

The Na^+^/H^+^ ion exchanger (NHE) pumps Na^+^ inward the cell and H^+^ ion outside the cell. NHE activity increases in response to a decrease in intracellular pH, and it maintains intracellular pH in a narrow range. Patients with obesity, diabetes, and hypertension and the elderly are prone to severe acute respiratory syndrome coronavirus-2 (SARS-CoV-2) infection. The angiotensin II (Ang II) level is high in chronic diseases such as diabetes, hypertension, and obesity. Ang II is the main stimulator of NHE, and an increased Ang II level causes prolonged NHE activation in these patients. The long-term increase in NHE activity causes H^+^ ions to leave the cell in patients with diabetes, hypertension, and obesity. Increasing H^+^ ions outside the cell lead to an increase in oxidative stress and reactive oxygen species. H^+^ ion flows into the cell due to the increased oxidative stress. This vicious circle causes intracellular pH to drop. Although NHE is activated when intracellular pH decreases, there is prolonged NHE activation in chronic diseases such as aforementioned. Novel coronavirus disease 2019 (COVID-19) progression may be more severe and mortal in these patients. SARS-CoV-2 readily invades the cell at low intracellular pH and causes infection. The renin-angiotensin system and NHE play a vital role in regulating intracellular pH. The reduction of NHE activity or its prolonged activation may cause susceptibility to SARS-CoV-2 infection by lowering intracellular pH in patients with diabetes, hypertension, and obesity. Prolonged NHE activation in these patients with COVID-19 may worsen the course of the disease. Scientists continue to investigate the mechanism of the disease and the factors that affect its clinical progression.

## 1. Introduction

Novel coronavirus disease 2019 (COVID-19) continues to spread worldwide. Besides, chronic diseases, such as diabetes, hypertension, and obesity, and aging increase susceptibility to severe acute respiratory syndrome coronavirus-2 (SARS-CoV-2) infection [1, 2]. COVID-19 progression may be critical and mortal in these patients [1]. Interestingly, the course of COVID-19 can be intense in a few young individuals and people with no chronic diseases [1]. For the virus to infect the cell, it must fuse with angiotensin-converting enzyme-2 (ACE2) [2]. A recent study reported that plasma ACE2 levels are higher in men than in women [3]; therefore, the disease is more severe in men than in women. ACE2 upregulation can increase the chance of the virus causing infection [4]. Besides, intracellular pH can play a crucial role in the virus-ACE2 fusion, increasing the susceptibility of patients with hypertension, diabetes, and obesity to the virus [5, 6]. The Na^+^/H^+^ ion exchanger (NHE), found in the membranes of many cells, is primarily responsible for maintaining intracellular pH and Na^+^ homeostasis [7]. There are nine subtypes of NHE, and the NHE1 type is the most common subtype. NHE plays a vital role in the cell cycle and many related functions and provides resistance to apoptosis. NHE ensures maintaining the electrolyte balance and pH by pumping Na^+^ into the cell and H^+^ outside the cell [7, 8]. In addition to ACE2, NHE may play a vital role in SARS-CoV-2 infection. Here, we will focus on the mechanisms affecting intracellular pH and the role of intracellular pH in SARS-CoV-2 infection. We will discuss whether ACE2 upregulation and prolonged NHE activation are beneficial or harmful in the COVID-19 progression. Also, through these mechanisms, we will discuss the effects of some drugs on susceptibility to SARS-CoV-2 and the course of COVID-19.

## 2. Entry of SARS-CoV-2

After SARS-CoV-2 enters the upper respiratory tract, it is transported to the lungs via respiration. When it reaches the lungs, it fuses with ACE2 in the membrane of these cells and infects the cell. During the fusion of the virus with ACE2, the intracellular pH level may play an important role [9].

## 3. Intracellular Compartments and Intracellular pH

The normal intracellular pH range is physiologically between 7.0 and 7.4, but there is variability between tissues [10–12]. Also, there is a pH difference between different organelles that can range from about 4.5 to 8.0 [10–12]. Intracellular compartments include endosomes, lysosomes, and Golgi vesicles [10]. Endosomes and lysosomes have a lumen that is more acidic than the cytoplasm [13]. Acidifying the lumen of these intracellular organelles plays a critical role in essential cellular processes. These basic cellular processes include autophagy, vesicle trafficking and fusion, lysosomal degradation, receptor-ligand interaction, and signaling [10, 13]. Thus, endosomes and lysosomes break down complex sugars, lipids, nucleic acids, and proteins with their hydrolase enzymes [10, 13]. Intracellular pH is acidic (for example, late endosome pH ~5.5–~6.0) [10], and intracellular acidity causes autophagy [5]. Although autophagy increases when intracellular pH decreases, SARS-CoV-2 can escape from autophagy by some mechanisms that have not been elucidated yet [14]. Therefore, acidic intracellular pH cannot prevent SARS-CoV-2 infection.

## 4. Intracellular pH and SARS-CoV-2

As the intracellular pH increases, autophagy decreases [5]. Hormones and peptides like angiotensin (Ang) II, vasopressin, norepinephrine, aldosterone, platelet-derived growth factor, epidermal growth factor, insulin, and certain adipokines raise intracellular pH [15–17]. High-salt diets and a low physiological concentration of 17*β*-estradiol cause intracellular pH alkalization [16, 18]. Decreased autophagy leads to susceptibility to viral and bacterial infections [5]. Since SARS-CoV-2 escapes autophagy, unlike other viruses, it invades the cell at low intracellular pH [14]. Low intracellular pH may facilitate the delivery of the virus genome to the cytosol [2]. Thus, further viral replication in the cytosol causes the creation of mature virions and subsequent spread [2, 19]. When the intracellular pH is alkaline, the risk of the virus infecting the cell decreases [3, 6].

## 5. NHE Regulates Intracellular pH

The NHE exists in the membrane of many cells and pumps Na^+^ inward and H^+^ outward [6, 20]. NHE and vacuolar ATPase (V-ATPase) regulate intracellular pH [5, 21]. Physiologically, increased NHE activity or V-ATPase inhibition increases intracellular pH [5, 21]. The Ca^2+^/H^+^ ion antiport activates simultaneously with NHE and pumps Ca^2+^ into the cell [9, 20]. NHE activity increases in response to a decrease in intracellular pH, and NHE protects the cell from acidification and maintains intracellular pH in a narrow range [21, 22].

## 6. Angiotensin II and NHE

Angiotensinogen, a peptide produced from the liver, is converted to Ang I by renin and Ang I to Ang II by the enzyme ACE [23]. Ang II increases Na^+^-H^+^ exchange, leading to Na^+^ reabsorption, the increased osmolarity of the blood, edema, and increased blood pressure. Ang II is the main stimulator of NHE and plays a key role in its regulation of intracellular pH [24]. Experimental acid loading models have shown that Ang II activates NHE, causing a sudden rise in intracellular pH and bringing intracellular pH to the normal range [24].

## 7. Hypertension, Diabetes, and Obesity and NHE

The renin-angiotensin system (RAS) is overactive in patients with hypertension, diabetes, and obesity, and their Ang II level was found to be high [25–27]. Angiotensinogen synthesis raises proportionally to the body mass index (BMI) [27]. Besides, there is a close inverse relationship between BMI and fasting intracellular pH [2]; insulin and adipokine release and Ang II stimulation increase intracellular pH by increasing NHE activity [15–17]. However, prolonged NHE activation by the mechanism discussed below causes a decrease in intracellular pH; as the BMI increases, insulin resistance and adipokine and Ang II production increase, so the intracellular pH is low in individuals with high BMI [28]. Therefore, patients with obesity are susceptible to COVID-19. NHE is physiologically activated when intracellular pH decreases in healthy individuals [29]. In healthy people, NHE pumps H^+^ out of the cell, bringing the pH from acidic to normal [29]. Insulin, glucose, norepinephrine, Ang II, aldosterone (which is increased in hypertension), and some adipokines (which are increased in diabetes and obesity) cause prolonged NHE activation in patients with hypertension, diabetes, and obesity [17, 20, 30, 31].

## 8. Prolonged NHE Activation and Oxidative Stress

Prolonged NHE activity causes H^+^ ions to move out of the cell. Increasing H^+^ ions outside the cell increase oxidative stress and reactive oxygen species (ROS) [20, 30]. H^+^ ion flows into the cell due to the increased oxidative stress [32]. Because of this vicious circle, in patients with hypertension, diabetes, and obesity, intracellular pH values are lower than in healthy individuals [30]. Elevated Ang II levels and activated NHE in these patients cause vasoconstriction and reduce tissue blood perfusion [30, 31]. Since it impairs tissue blood perfusion, ROS increases, and acid-base disturbances occur [30, 31]. Besides, an increase in Na^+^ and Ca^2+^ levels within the cell causes edema and triggers apoptosis. Increased NHE activation damages the vascular endothelium, cells, and tissues in patients with hypertension, diabetes, and obesity [33].

## 9. Elderly and NHE

The virus attaches to ACE2 and infects cells [34]. The RAS is more active in young people, and their ACE2 levels are higher than in the elderly, and their intracellular pH is in the normal range [35]. Therefore, they are not as susceptible to SARS-CoV-2 infection as the elderly. When the NHE activity is reduced or blocked, the intracellular pH drops by about 0.6 pH units, and NHE activity and intracellular pH may decrease in the elderly [36–38]. Older people are more prone to SARS-CoV-2 infection than younger people since low intracellular pH can play a role in SARS-CoV-2 entry into the cell [1, 25].

## 10. SARS-CoV-2 and Prolonged NHE Activation

Prolonged NHE activation, not physiological NHE activation, causes a decrease in intracellular pH [20, 30]. NHE activation and H^+^ influx depend on the extracellular Na^+^ concentration, and a drop of 0.6 pH units in intracellular pH results in H^+^ being pumped out of the cell up to 24-fold [29, 36]. The elderly and patients with diabetes, hypertension, and obesity who have low intracellular pH may be more susceptible to SARS-CoV-2 since the total amount of H^+^ produced in these patients is much larger than that in healthy persons [25, 39]. Endosomal alkalization reduces the fusion event between SARS-CoV-2 and ACE2 by leading to ACE2 downregulation or ACE2 glycosylation [5, 25].

## 11. Interaction among NHE, ACE2, and SARS-CoV-2

In COVID-19 patients, the fusion of the ACE2 with the virus may cause ACE2 to be dysfunctional. This ACE2 cannot convert Ang II to Ang 1-7, causing an increased Ang II level and prolonged NHE activity [33, 34]. In COVID-19 patients, intracellular accumulation of Na^+^ and Ca^2+^ and increased oxidative stress cause damage to many organs, which have ACE2, such as the lung, endothelium, heart, liver, and pancreas [20, 33]. Due to this damage, the hospitalization period and death risks of patients with COVID-19 may increase. Increased RAS activity and ACE upregulation downregulate ACE2, vice versa. The Ang II increase or decrease affects the intracellular pH and COVID-19 course. Therefore, the balance between RAS, ACE, and ACE2 is vital for COVID-19 [40].

## 12. Drugs Interact with ACE2 and NHE

Patients with hypertension, diabetes, and obesity frequently use glucagon-like peptide-1 receptor (GLP-1R) agonists, angiotensin receptor blockers (ARBs), and ACE inhibitors (ACEIs). These drugs cause ACE2 upregulation and prevent prolonged NHE activation in patients with hypertension, diabetes, and obesity [41, 42]. They create a beneficial effect by ensuring the excretion of Na^+^ through the kidneys, reducing the production of excess H^+^ and oxidative stress [41, 42]. However, ACEIs, ARBs, and GLP-1R agonists may cause susceptibility to SARS-CoV-2 infection due to ACE2 upregulation [43]. Many studies reported that ARBs and ACEIs do not inversely affect the mortality rate and hospital stay of patients with COVID-19 [44]. ACEIs and ARBs may reduce mortality in COVID-19, according to recent studies [45]. Health authorities recommended that patients continue to use these drugs during COVID-19 [45]. A previous study speculated that GLP-1R agonists did not contribute to the recovery of patients with COVID-19 [46]. On the other hand, GLP-1R agonist-associated ACE2 respiratory upregulation is infrequent and shown in few animal studies only [47]. GLP-1R agonists have powerful glucose-lowering and weight loss effects [2, 47]. Besides, they may have anti-inflammatory and pulmonary protective effects and a beneficial impact on gut microbiome composition. Because of these effects, GLP-1R agonists can be helpful in the treatment of diabetic, hypertensive, and obese people with COVID-19 [47].

## 13. Are Drugs Harmful or Useful?

Inhibition of prolonged NHE activity and ACE2 upregulation can also benefit COVID-19 patients. While NHE inhibition maintains intracellular pH within the normal range, ACE2s, which do not fuse with the virus, remain functional. Although Ang 1-7, the product of ACE2, does not affect intracellular pH [25], GLP-1R agonists, ACEIs, and ARBs can reduce the progression and damage of COVID-19 by causing a vasodilator and antioxidant effect through activation of the ACE2/Ang 1-7/Mas receptor pathway [2]. Also, activation of this pathway stimulates the immune system and may increase host defense against SARS-CoV-2 [48]. On the other hand, drugs that cause ACE2 upregulation can be defined as a double-edged sword, as they can be beneficial or harmful in SAR-CoV-2 infection [43]. GLP-1R agonists, ACEIs, and ARBs may create a backdoor with ACE2 upregulation, making it easier for the virus to occupy cells [43]. If the virus load is high and there is ACE2 upregulation, the viruses can easily infect the cells by fusing with ACE2 since there is more ACE2 in the cells. However, these drugs that caused ACE2 upregulation may be beneficial in patients with COVID-19 if the virus remains in a restricted organ such as the lung. The drugs can prevent oxidative damage and maintain the electrolyte balance of cells by inhibiting NHE during the virus infection.

## 14. Drug Beneficial Effects

Scientists do not yet know whether ACEIs and ARBs are superior to each other concerning beneficial effects in COVID-19. While ACEIs decrease the ACE level [40], they increase the bradykinin level [49]. Increased bradykinin alkalizes the intracellular pH [49]. A recent study reported a 40% reduction in hospitalization rates in elderly patients with COVID-19 using ACEIs; however, this effect was not observed in elderly patients using ARBs [50]. Inhibition of prolonged NHE activity may cause intracellular pH to change from acidic to normal, making it difficult for SARS-CoV-2 to fuse with ACE2. Additionally, inhibition of prolonged NHE leads to a reduction in oxidative stress and may prevent damage to cells and tissues in patients with COVID-19. Therefore, choosing ACEIs instead of ARBs in patients with COVID-19 may benefit patients.

## 15. Conclusion

The RAS and NHE activities play a vital role in regulating intracellular pH. Prolonged NHE activation may cause susceptibility to SARS-CoV-2 infection by lowering the intracellular pH of patients with hypertension, diabetes, and obesity. Prolonged NHE activation in these patients with COVID-19 may worsen the course of the disease by causing oxidative stress and cell damage. We believe that prolonged activation of NHE increases susceptibility to SARS-CoV-2 and affects COVID-19 severity. Besides, ACEIs, ARBs, and GLP-1R agonists cause ACE2 upregulation, NHE inhibition, and changed intracellular pH. ACEIs, ARBs, and GLP-1R agonists may lead to many beneficial effects in patients with hypertension, diabetes, and obesity during COVID-19 by ACE2 upregulation and NHE inhibition. However, ACE2 upregulation may increase susceptibility to SARS-CoV-2 in these patients. Further well-designed clinical and experimental studies are needed to draw final conclusions.
